# Pre-Pregnancy BMI and Gestational Weight Gain Have Independent Associations with Birth Weight: A Prospective Cohort Study in Mongolia

**DOI:** 10.3390/jcm15103638

**Published:** 2026-05-09

**Authors:** Nomin-Erdene Minjuurdorj, Enkhtsetseg Jamsranjav, Orgil Zorigtbaatar, Nasantsengel Lkhagvasuren, Oyuntugs Byambasukh, Altaisaikhan Khasag

**Affiliations:** 1 Department of Endocrinology, School of Medicine, Mongolian National University of Medical Sciences, Ulaanbaatar 14210, Mongolia; amd21f005@gt.mnums.edu.mn; 2 Department of Obstetrics and Gynecology, School of Medicine, Mongolian National University of Medical Sciences, Ulaanbaatar 14210, Mongolia; enkhtsetseg@mnums.edu.mn; 3 SOD MED Mental Health Center, Ulaanbaatar 16062, Mongolia; amd17f002@gt.mnums.edu.mn (O.Z.); mhcnasaa@gmail.com (N.L.)

**Keywords:** pre-pregnancy, body mass index, gestational weight gain, neonatal birth weight, independent association

## Abstract

**Background:** Neonatal birth weight is a key determinant of short- and long-term health outcomes. Pre-pregnancy body mass index (BMI) and gestational weight gain (GWG) are important predictors of birth weight; however, their independent associations remain unclear, particularly in settings where national GWG guidelines are lacking. **Methods:** A prospective cohort study was conducted among 340 pregnant women in Ulaanbaatar, Mongolia in 2022. Data were collected using standardized questionnaires and anthropometric measurements. Pre-pregnancy BMI and GWG were classified according to World Health Organization criteria and the 2009 Institute of Medicine guidelines. Multivariable linear regression models were used to examine independent associations with neonatal birth weight. **Results:** The mean maternal age, pre-pregnancy BMI, and GWG were 30.3 ± 6.0 years, 23.5 ± 4.4 kg/m^2^, and 14.3 ± 6.2 kg, respectively. Both BMI and GWG were independently associated with neonatal birth weight (*p* < 0.001), with no significant interaction observed (*p* = 0.147). Birth weight increased by 29.7 g (95% CI: 18.6–40.8) per 1 kg/m^2^ increase in BMI and by 31.7 g (95% CI: 24.0–39.4) per 1 kg increase in GWG. Compared with normal BMI, overweight/obesity was associated with higher birth weight, while excessive GWG increased and inadequate GWG decreased birth weight (all *p* < 0.05). **Conclusions:** Pre-pregnancy BMI and GWG were independently associated with neonatal birth weight, with GWG showing a slightly stronger association. These findings highlight the importance of optimizing maternal weight before and during pregnancy. Countries undergoing rapid nutritional transitions may benefit from developing context-specific GWG guidelines, with Mongolia representing a relevant example.

## 1. Introduction

Neonatal birth weight is a key indicator of intrauterine growth and an important predictor of neonatal morbidity, childhood development, and long-term health outcomes [1,2,3,4]. Both low birth weight (LBW) and macrosomia are associated with increased perinatal complications. LBW, defined as less than 2.5 kg [5], is a significant public health concern worldwide and a leading cause of neonatal mortality, primarily due to prematurity, intrauterine growth restriction, and increased risk of neurodevelopmental impairment, with lasting consequences for growth and development [1,2,4]. In contrast, macrosomia (birth weight ≥ 4000 g) is associated with not only delivery-related complications in the newborn, such as shoulder dystocia and birth trauma but also maternal complications, including cesarean delivery and hypertensive disorders of pregnancy. It may further predispose individuals to long-term metabolic consequences, such as obesity, insulin resistance, and cardiovascular disease later in life [3,6,7,8].

Maternal nutritional status before and during pregnancy plays a central role in determining fetal growth [9,10]. Pre-pregnancy body mass index (BMI) reflects maternal nutritional and metabolic status at conception, whereas GWG represents the cumulative physiological and nutritional changes throughout pregnancy. Numerous studies have reported associations between higher pre-pregnancy BMI, excessive GWG, and increased neonatal birth weight, while inadequate GWG has been linked to fetal growth restriction [8,11,12,13,14]. To address these risks, the Institute of Medicine (IOM) established guidelines in 1990, which were updated in 2009, to provide BMI-specific recommendations for weight gain [15]. These guidelines are widely adopted in clinical practice and research worldwide [14,15].

Despite the inherent correlation between pre-pregnancy BMI and GWG, separating their individual association on neonatal birth weight remains a persistent challenge. While maternal BMI and GWG are extensively studied in relation to pregnancy complications such as gestational diabetes, preterm birth, and hypertensive disorders of pregnancy [7,12,13,14,16,17], their isolated impact on birth weight alone is less clearly defined [11,18,19]. Such inconsistency leaves a critical gap in our understanding of whether these factors influence fetal growth through distinct biological pathways or shared mechanisms, particularly across diverse populations [20].

Mongolia, a landlocked nation of 3.5 million people with 842,427 women of reproductive age, has undergone a swift socio-demographic transformation over the past few decades. A significant portion of the population has shifted from traditional nomadic and semi-nomadic lifestyles toward urbanized living, with nearly half of the nation’s inhabitants now residing in the capital city, Ulaanbaatar [21]. This rapid and concentrated urbanization has been linked to an increase in metabolic risk factors for non-communicable diseases; for instance, the prevalence of overweight and obesity in women has increased from 38% in 2005 to 50.5% in 2019 [22,23,24,25]. Concurrently, the national birth rate has declined from 77,710 in 2020 to 59,644 in 2024 [26]. In the context of this declining birth rate and shifting demographics, protecting optimal neonatal health as measured primarily by birth weight has become a critical health priority for the nation.

While the association of pre-pregnancy BMI and GWG on neonatal outcomes is extensively documented globally, studies examining their independent association within the same analytical framework remain limited. Although the Ministry of Health has established prenatal care guidelines based on World Health Organization (WHO) recommendations [27], specific national standards for GWG have not yet been defined. In the absence of local standards, this study adopts the globally recognized Institute of Medicine (IOM) guidelines, facilitating direct comparisons with international literature. Furthermore, by managing these modifiable risk factors through cost-effective clinical interventions, it is possible to enhance neonatal outcomes and work toward the strategic goal of reducing maternal and infant morbidity. Conducting this research in Mongolia serves as a valuable reference for other developing countries with comparable healthcare systems, providing practical insights applicable to similar clinical settings. Therefore, this study aimed to examine the independent associations of pre-pregnancy BMI and gestational weight gain with neonatal birth weight in a Mongolian population using mutually adjusted multivariable regression models.

## 2. Materials and Methods

### 2.1. Study Design and Participants

**Figure 1:**
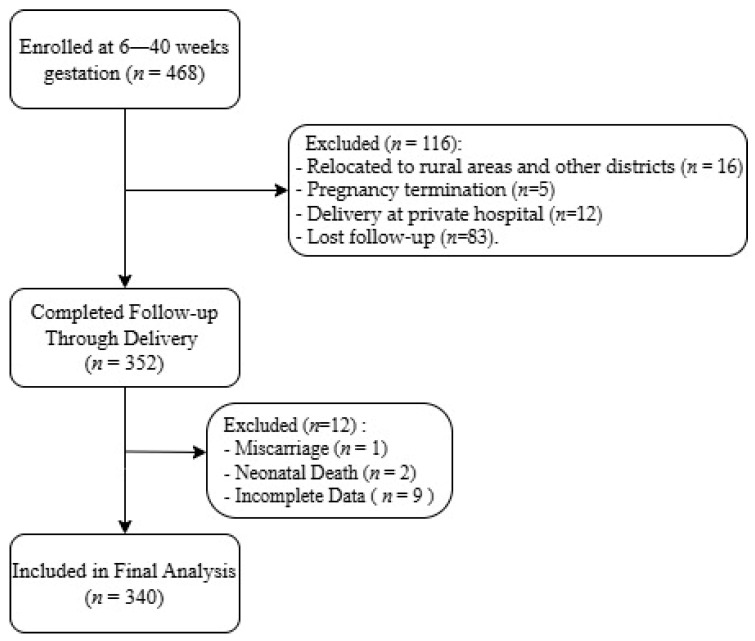
Flow chart of the study.

This prospective cohort study was conducted at two district health centers (Bayanzurkh and Chingeltei) in Ulaanbaatar, Mongolia in 2022. The study was approved by the Ethics Committee of the Mongolian National University of Medical Sciences (Approval No. 2022/3-02, date of 18 February 2022). All participants provided written informed consent in the Mongolian language prior to enrollment. Pregnant women attending routine antenatal care were consecutively recruited between 21 February and 10 March 2022 and followed from enrollment until delivery. All eligible pregnant women attending antenatal care at the study sites during the recruitment period were invited to participate. Initially, 468 pregnant women with gestational ages between 6 and 40 weeks were enrolled. During the follow-up period, participants were excluded due to relocation to rural areas or other districts (*n* = 16), pregnancy termination (*n* = 5), delivery at private hospitals (*n* = 12), and loss to follow-up (*n* = 83). Of these, 352 completed follow-ups through delivery. After excluding participants with miscarriage (*n* = 1), neonatal death (*n* = 2), and incomplete data (*n* = 9), 340 women were included in the final analysis. The flow chart of participants through the study is presented in Figure 1. Inclusion criteria were Mongolian citizenship, age ≥ 18 years, singleton pregnancy, and absence of pre-existing chronic diseases. Women who declined to participate in the study or had multiple pregnancies were excluded.

### 2.2. Data Collection

A face-to-face interview collected data using structured questionnaires on socio-demographic characteristics, obstetric history, current pregnancy information, and general health, including alcohol use and smoking habits. The questionnaires were based on the publicly available STEPS 2019 survey report and taken from the Mongolian language appendices [25]. Data were collected by trained researchers, including medical doctors, using a single, standardized protocol to ensure uniformity and minimize potential bias. Pre-pregnancy weight was obtained from antenatal health records documented at the first antenatal visit. Maternal height was measured during the first antenatal visits using standard measurement techniques. According to the WHO criteria, pre-pregnancy BMI was categorized as underweight (<18.5 kg/m^2^), normal weight (18.5–24.9 kg/m^2^), overweight (25.0–29.9 kg/m^2^), and obese (≥30.0 kg/m^2^). Participants were followed until delivery. Maternal weight at delivery was obtained from maternity hospital medical records, while pre-pregnancy weight was documented in the antenatal health card. Total GWG was calculated using the following formula:

GWG = Weight_delivery_ − Weight_pre-pregnancy_

Participants were categorized into inadequate, adequate, and excessive GWG groups based on the 2009 Institute of Medicine (IOM) guidelines (Table 1) [28].

Neonatal birth weight data were obtained from maternity hospital official medical records. Newborns were weighed within one hour of birth using electronic scales with an accuracy of ±10 g. Newborn birth weight was categorized as low birth weight (<2500 g) [5], normal birth weight (2500–3999 g), and macrosomia (≥4000 g) [6]. Gestational age at delivery was calculated in days from the first day of the last menstrual period and categorized into two groups: preterm birth, defined as delivery before 37 completed weeks of gestation (<259 days), and term birth, defined as delivery at 37 completed weeks or later (≥259 days) [29].

### 2.3. Statistical Analysis

Continuous variables were presented as mean ± standard deviation (SD), and categorical variables were expressed as number (percentage). Differences in maternal characteristics across newborn birth weight categories (low birth weight, normal birth weight, and macrosomia) were evaluated using one-way analysis of variance (ANOVA) for continuous variables and Pearson’s chi-square test for categorical variables, with Fisher’s exact test applied for small cell frequencies (<5).

Pearson correlation analysis was used to examine the relationships between pre-pregnancy BMI, total GWG, and newborn birth weight. An interaction term between pre-pregnancy BMI and GWG was added to evaluate their combined association on newborn birth weight.

Linear regression analyses were conducted to assess the association of pre-pregnancy BMI and GWG with newborn birth weight. In the unadjusted model, BMI categories (underweight, normal, overweight/obese) and GWG categories (inadequate, adequate, excessive) were included as independent variables. Normal BMI and adequate GWG were used as reference categories. Multivariable linear regression models were then constructed. The first adjusted model included general maternal characteristics (age, education level, smoking status, and alcohol consumption). The further models were adjusted for gestational characteristics. Beta coefficients (β) and 95% confidence intervals (CIs) were reported.

All statistical analyses were performed using IBM SPSS version 28.0 (IBM Corp., Armonk, NY, USA). A *p*-value of <0.05 was considered statistically significant for all analyses.

## 3. Results

### 3.1. General Characteristics of the Study Population

A total of 340 pregnant women were included in the analysis. The mean maternal age was 30.26 ± 5.97 years. Most participants were married (70.9%), had higher education (58.8%), were employed (69.1%), and were of Khalkh ethnicity (87.4%). The mean parity was 1.54 ± 1.25, and the mean gravidity was 3.12 ± 1.69, with 72.9% being multiparous. Smoking was reported by 3.2% of women, and 27.4% reported alcohol consumption.

The mean pre-pregnancy BMI was 23.48 ± 4.40 kg/m^2^. Based on BMI classification, 9.4% (*n* = 32) were underweight, 59.4% (*n* = 202) had normal BMI, 22.7% (*n* = 77) were overweight, and 8.5% (*n* = 29) were obese. Regarding GWG, 28.2% (*n* = 96) had inadequate gain, 32.9% (*n* = 112) had adequate gain, and 38.9% (*n* = 132) had excessive gain. The mean GWG was 14.31 ± 6.16 kg.

Neonatal outcomes showed that 2.9% (*n* = 10) of newborns had low birth weight (<2500 g), 84.7% (*n* = 288) had normal birth weight (2500–3999 g), and 12.4% (*n* = 42) had macrosomia (>4000 g).

The mean gestational age at delivery was 274.21 ± 10.60 days, and it was lower in the low-birth-weight group (258.20 ± 20.07 days) compared with the normal birth weight (274.74 ± 10.07 days) and macrosomia groups (274.33 ± 8.15 days) (*p* < 0.001).

In terms of gestational age ate delivery classification, preterm birth (<259 days/<37 weeks) accounted for 7.9% (*n* = 27) of the total participants. This proportion was substantially higher in the low-birth-weight group 40.0% (*n* = 4), while 7.3% (*n* = 21) in the normal birth weight group and 4.8% (*n* = 2) in the macrosomia group. The majority of deliveries occurred at term (≥259 days), representing 92.1% (*n* = 313) overall, including 60.0% in the low-birth-weight group, 92.7% in the normal birth weight group, and 95.2% in the macrosomia (Table 2).

### 3.2. Maternal Characteristics by Birth Weight Categories

Table 2 presents maternal characteristics stratified by newborn birth weight categories. Maternal age, age group distribution, marital status, employment status, ethnicity, gravidity, and maternal height did not differ significantly across birth weight groups (all *p* > 0.05).

Education level was significantly associated with birth weight categories (*p* = 0.046). Although mean parity did not differ significantly (*p* = 0.138), parity categories were significantly associated with birth weight (*p* = 0.003), with multiparity more common among women delivering macrosomic infants. Smoking status and alcohol consumption also differed significantly across birth weight groups (*p* = 0.007 and *p* = 0.022, respectively).

Pre-pregnancy weight, pre-pregnancy BMI, total GWG, and maternal BMI before delivery were significantly higher among women delivering macrosomic infants (all *p* < 0.001).

### 3.3. Birth Weight Distribution by Pre-Pregnancy BMI and GWG

**Figure 2:**
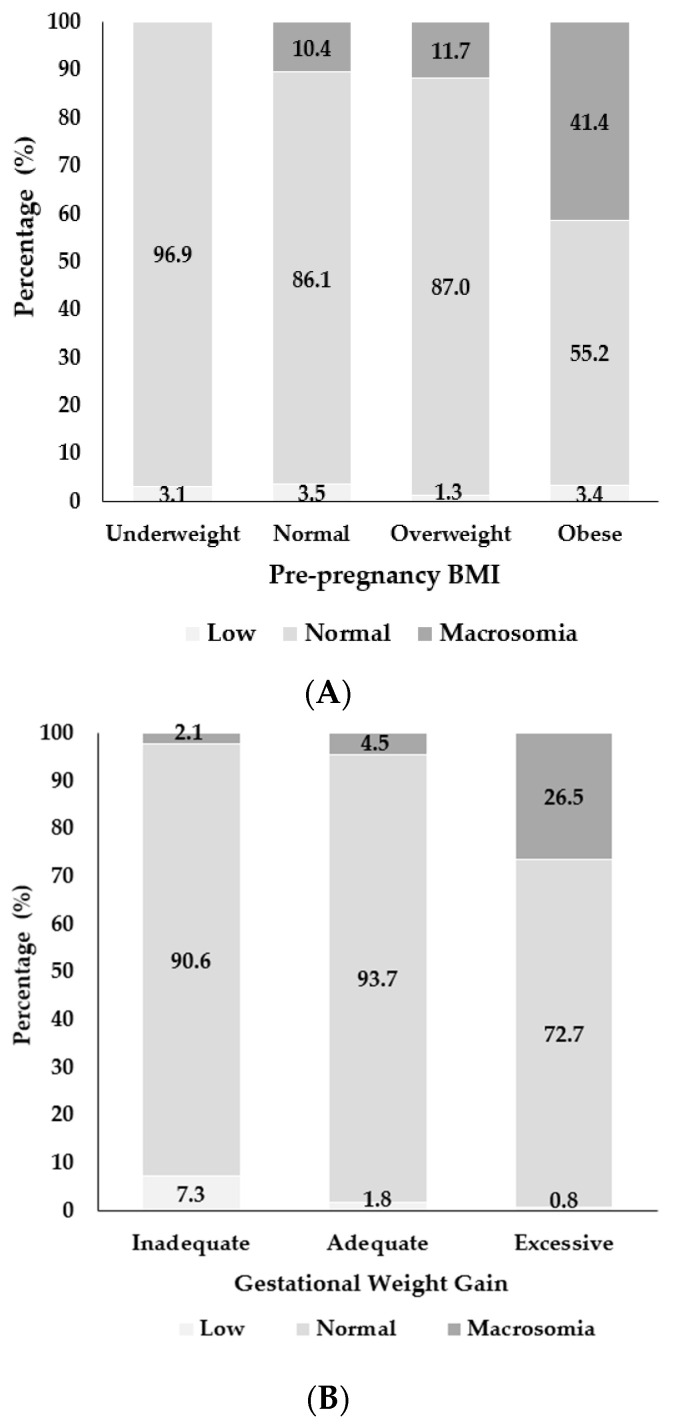
(**A**) Newborn birth weight by pre-pregnancy BMI categories. (**B**) Newborn birth weight by GWG categories.

Figure 2A shows newborn birth weight distribution by pre-pregnancy BMI categories. The proportion of macrosomia increased progressively with higher BMI, rising from 10.4% in the normal-weight group to 41.4% among women with obesity—representing a nearly four-fold increase in prevalence. While normal birth weight predominated among women with normal (86.1%) and overweight (87.0%) BMI, the sharp rise in the obese category highlights maternal obesity as a major clinical determinant of macrosomia. Low birth weight remained infrequent across all BMI categories.

Figure 2B presents birth weight distribution according to GWG categories. Macrosomia was most common among women with excessive GWG (26.5%), whereas low birth weight was more frequent among those with inadequate GWG (7.3%).

**Figure 3:**
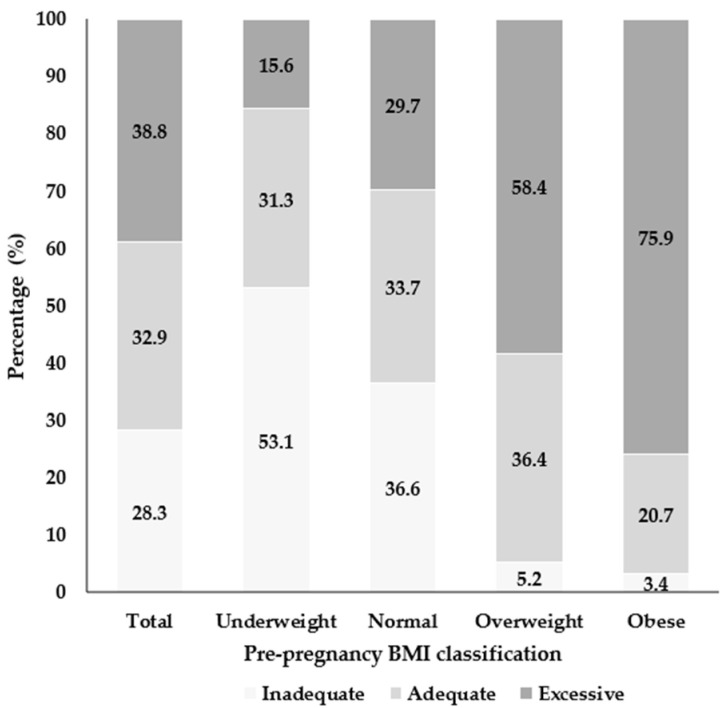
GWG by IOM classification in pre-pregnancy BMI categories.

Figure 3 illustrates GWG according to IOM recommendations across pre-pregnancy BMI categories. Excessive GWG was particularly prevalent among overweight (58.4%) and obese women (75.9%), whereas inadequate GWG was most common among underweight women (53.1%).

### 3.4. Multivariable Linear Regression Analysis (Continuous Variables)

In a multivariable linear regression model including pre-pregnancy BMI and GWG as continuous variables and adjusted for maternal age, education, smoking, alcohol use, and parity, both variables remained independently associated with neonatal birth weight.

Each 1 kg/m^2^ increase in pre-pregnancy BMI was associated with a 29.69 g increase in birth weight (95% CI: 18.61 to 40.76; *p* < 0.001). Similarly, each 1 kg increase in GWG was associated with a 31.66 g increase in birth weight (95% CI: 23.96 to 39.36; *p* < 0.001). These effect sizes indicate that both pre-pregnancy BMI and gestational weight gain have clinically meaningful and independent contributions to neonatal birth weight. None of the covariates were significantly associated with birth weight in this model.

### 3.5. Correlation and Interaction Analysis

**Figure 4:**
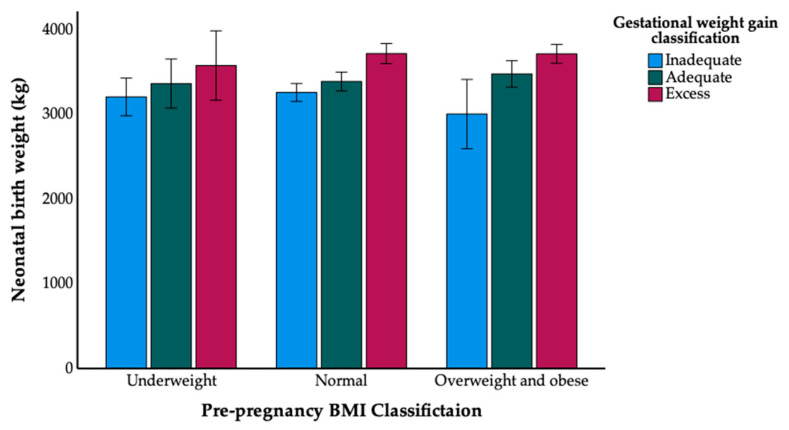
Means of birth weight by pre-pregnancy BMI and gestational weight gain (IOM classification).

Correlation analysis demonstrated that both pre-pregnancy BMI (r = 0.255, *p* < 0.001) and GWG (r = 0.388, *p* < 0.001) were positively correlated with neonatal birth weight. However, the interaction term between pre-pregnancy BMI and GWG was not statistically significant (β = −1.43, *p* = 0.147). The non-significant interaction term indicates that the association between gestational weight gain and birth weight does not differ across levels of pre-pregnancy BMI. This is further supported by the means plot (Figure 4), which demonstrates parallel increases in birth weight across GWG categories within each BMI group.

### 3.6. Categorical Regression Models

Table 3 presents regression analyses using categorical BMI and GWG variables. In the unadjusted model, overweight/obese women delivered infants with significantly higher birth weight compared with women with normal BMI (β = 166.14; 95% CI: 48.92 to 282.84; *p* = 0.005). Inadequate GWG was associated with lower birth weight (β = −176.86; 95% CI: −303.44 to −50.28; *p* = 0.006), whereas excessive GWG was associated with higher birth weight (β = 297.26; 95% CI: 180.34 to 414.17; *p* < 0.001). After adjustment for general characteristics, these associations remained similar.

In the model adjusted for gestational characteristics, overweight/obesity remained significantly associated with increased birth weight (β = 179.39; 95% CI: 87.07 to 277.76; *p* < 0.001). Inadequate GWG remained negatively associated (β = −152.75; 95% CI: −281.25 to −24.24; *p* = 0.020), and excessive GWG remained positively associated with birth weight (β = 257.07; 95% CI: 136.62 to 377.19; *p* < 0.001). Finally, in the model adjusted for gestational age at delivery, overweight/obesity group remained significantly associated with increased birth weight (β = 155; 95% CI: 39.1 to 272.1; *p* = 0.009). Inadequate GWG remained negatively associated with birth weight (β = −176.7; 95% CI: −302.3 to −51.1; *p* = 0.006), and excessive GWG remained positively associated with birth weight (β = 281.4; 95% CI: 164.8 to 398.1; *p* < 0.001).

## 4. Discussion

In this study, we investigated the independent associations of pre-pregnancy BMI and GWG on neonatal birth weight using mutually adjusted regression models. A key finding is that both maternal BMI and GWG were independently associated with neonatal birth weight. Notably, maternal underweight and inadequate GWG were associated with lower neonatal birth weight, whereas maternal obesity and excessive GWG were associated with higher neonatal birth weight. Furthermore, our analysis indicates that GWG is a critical modifiable determinant of birth weight regardless of pre-pregnancy BMI, as evidenced by the absence of a significant interaction between BMI and GWG. This suggests that pre-pregnancy BMI and gestational weight gain influence fetal growth through largely independent biological pathways. Pre-pregnancy BMI reflects baseline maternal metabolic status, including insulin resistance and adiposity prior to conception, which may influence early placental development. In contrast, gestational weight gain reflects dynamic metabolic adaptations during pregnancy, particularly in the second and third trimesters, affecting nutrient availability and transplacental transfer. These distinct temporal and physiological mechanisms may explain why their effects on birth weight are additive rather than multiplicative. These findings highlight the need to formally incorporate GWG targets into prenatal care guidelines.

In previous studies, Hull et al. and Sewell et al. have reported that maternal obesity and excessive gestational weight gain are associated with increased neonatal fat mass and birthweight, which is consistent with our findings [30,31,32]. These associations can be explained by biological pathways involving hormonal regulation, metabolic adaptation during pregnancy, and altered placental nutrient transport. Excessive gestational weight gain reflects metabolic changes during pregnancy, leading to increased nutrient transfer from the mother to the fetus. The placenta plays a central role in supporting fetal growth by transferring nutrients from the women to the fetus [33]. During the second and third trimesters, the placenta secretes insulin-antagonistic hormones such as human placental lactogen (hPL), estrogen, progesterone, placental growth hormone (PGH), and cortisol, contributing to increased maternal insulin resistance [6,34]. As a result, maternal insulin resistance is further exacerbated, leading to metabolic disturbances such as hyperglycemia, dyslipidemia, and altered adipokine profiles, including reduced adiponectin levels and increased pro-inflammatory cytokines [35,36,37,38,39,40,41]. These pregnancy-related metabolic disturbances promote excessive transplacental nutrient transfer, thereby increasing fetal growth and adiposity, ultimately resulting in higher birth weight.

Pre-pregnancy obesity is associated with baseline insulin resistance and chronic metabolic disturbances, which are further exacerbated by pregnancy-related hormonal changes, leading to increased maternal glucose and lipid levels [41,42,43,44]. This metabolic environment promotes increased transplacental transport of glucose, amino acids, and lipids to the fetus [6,34,38]. Since maternal insulin does not cross the placental barrier, excess glucose is transported via GLUT1, resulting in fetal hyperglycemia and subsequent fetal hyperinsulinemia, which acts as a key anabolic driver of fetal growth [34]. Simultaneously, the excessive supply of transported amino acids and lipids serves as the essential substrate for stimulating gluconeogenesis, lipogenesis, and anabolism, which directly leads to increased adipose tissue accumulation and macrosomia [34]. These metabolic processes ultimately result in sustained fetal overgrowth.

The IOM guidelines recommend an inverse relationship between pre-pregnancy BMI and GWG [28]. However, GWG in our study population deviated from these recommendations: 53.1% of underweight women had inadequate GWG, while 75.9% of obese women exceeded recommended targets. Similar patterns have been reported in South Korea and China, where women with lower BMI often gain insufficient weight and those with higher BMI frequently exceed guidelines [45,46]. Compared with global trends reported by Goldstein et al. (2018, 2025) [12,16], the prevalence of inadequate GWG among underweight women in our study was higher than the 2025 estimate (27%), but similar to the 2018 estimate (43%). In contrast, excessive GWG among obese women remained relatively stable at approximately 60% in both years, consistent with our findings [12,16]. Additionally, our results regarding insufficient weight gain align with a sub-cohort analysis from Saudi Arabia, which reported that 59.9% of underweight women experienced inadequate GWG [17]. This variation may reflect population-specific physiological and ethnic differences, lifestyle factors, and the lack of standardized clinical counseling on optimal GWG.

GWG is not only associated with neonatal birth weight but also serves as a critical modifiable determinant of maternal and fetal complications during pregnancy and childbirth [12,13,20,45]. In our study, GWG showed the strongest and most consistent associations with neonatal birth weight. Compared with adequate weight gain, inadequate gain was associated with a reduction in birth weight of approximately 153 g, while excessive gain led to an increase of about 257 g. These findings indicate that deviations from recommended weight gain had a significant association on fetal growth. Similar associations between GWG and neonatal birth weight have been reported in previous studies by Uchinuma et al. [47], Wang et al. [48], and Ludwig and Currie [18]. In addition, findings from the BRISA cohort study suggested that GWG had stronger impact on birth weight than pre-pregnancy BMI [11]. Although public health attention has focused on excessive weight gain and its consequences, our results highlight that inadequate GWG also remains an important risk factor for lower neonatal birth weight. From a clinical perspective, this underscores the importance of monitoring not only excessive but also insufficient weight gain during pregnancy.

Pre-pregnancy BMI also demonstrated meaningful associations with neonatal birth weight. Compared with normal BMI, maternal overweight and obesity before pregnancy were associated with higher neonatal birth weight. This finding is consistent with previous studies by Yang et al. in China [49] and Enomoto et al. in Japan [50], which demonstrate that higher BMI categories are positively associated with increased infant birth weight. Although maternal underweight showed a downward trend in birth weight, the lack of statistical significance likely reflects limited power due to the small sample size of this group. Nonetheless, the observed trend reinforces the relevance of preconception nutritional status for fetal growth. These results align with previous studies by Liu et al. [14] and Yu et al. [19], which highlight preconception BMI as a more consistent predictor of neonatal outcomes than GWG alone. Therefore, focusing on maternal pre-pregnancy weight may be more effective in improving neonatal birthweight than relying solely on weight management during pregnancy.

This study has several strengths. The use of categorical exposures allowed for the examination of both insufficient and excessive maternal weight status. The application of mutually adjusted regression models enabled the estimation of independent associations while avoiding overadjustment. Furthermore, adjustment for key sociodemographic and behavioral factors strengthens the validity of the findings. However, several limitations should be acknowledged. First, as the study was conducted in only two districts of Ulaanbaatar and applied specific exclusion criteria, the findings may not be fully representative of the broader population of reproductive-age women in Mongolia. Second, although the reasons for follow-up loss were documented, the final sample size was reduced to 340 participants. This reduction not only limited the feasibility of a post hoc analysis to include initially eligible participants but also reduced the statistical power to detect smaller effect sizes in certain subgroup analyses, such as those for low-birth-weight infants or underweight women. Furthermore, the wide range of gestational ages at enrollment may have introduced variability in the assessment of gestational weight gain. Additionally, although pre-pregnancy weight was obtained from health records, it was based on maternal self-reported weight at the first antenatal visit, which may introduce measurement error. Finally, although we adjusted for several key sociodemographic factors, data on infant sex, an important confounding factor, were not available in the dataset, and we were also unable to fully account for other lifestyle variables, such as detailed dietary intake or physical activity levels, which may have resulted in residual confounding.

Despite these limitations, the findings have important clinical implications regarding both preconception and gestational care. In the context of Mongolia’s rapid socio-demographic transition, the study supports the potential applicability of international Institute of Medicine (IOM) guidelines in similar resource-limited settings with comparable healthcare systems, while also providing a preliminary evidence base for developing evidence-based recommendations on gestational weight gain (GWG). From a preventive perspective, maternal health strategies should prioritize improving health education among women of reproductive age to achieve optimal preconception body mass index (BMI). During pregnancy, individualized counseling on appropriate gestational weight gain, balanced nutrition, and physical activity should be routinely integrated into clinical practice. The implementation of standardized GWG guidelines may facilitate cost-effective clinical interventions and routine monitoring, thereby contributing to the prevention of both excessive and inadequate gestational weight gain, as well as associated adverse obstetric outcomes, including abnormal birth weight. Future research should focus on large-scale, multi-center studies and long-term cohort follow-up to further elucidate the long-term health consequences for both mothers and their offspring.

## 5. Conclusions

In this study, both pre-pregnancy BMI and GWG were independently associated with neonatal birth weight, with GWG exerting a more pronounced association. Maternal overweight or obesity before pregnancy and excessive GWG were linked to higher neonatal birth weight, whereas inadequate GWG was associated with lower birth weight. Importantly, our findings emphasize that prenatal care must address the full spectrum of weight issues, highlighting that interventions should focus not only on preventing excessive weight gain but also on avoiding insufficient gain. This underscores the critical necessity of integrating specific gestational weight gain targets, alongside pre-pregnancy BMI, into clinical guidelines to optimize maternal and neonatal outcomes.

## Figures and Tables

**Table 1:** Institute of Medicine (IOM) recommendations for total GWG according to pre-pregnancy BMI.

Weight Category	BMI Category Criteria	Recommended Weight Gain
Underweight	<18.5 kg/m^2^	12.5–18.0 kg
Normal weight	18.5–24.9 kg/m^2^	11.5–16 kg
Overweight	25.0–29.9 kg/m^2^	7–11.5 kg
Obese	≥30.0 kg/m^2^	5–9 kg

**Table 2:** Maternal characteristics by birth weight categories.

Variables		Total (*n* = 340)	Newborn Birth Weight, g			*p*-Value
			Low, <2500 (*n* = 10)	Normal, 2500–3999 (*n* = 288)	Macrosomia, >4000 (*n* = 42)	
Mean age (years)		30.26 ± 5.97	30.00 ± 9.33	30.23 ± 5.86	30.55 ± 5.95	0.941
Age group						
	<29	146 (42.9)	5 (50.0)	124 (43.1)	17 (40.5)	0.683
	30–34	101 (29.7)	1 (10.0)	86 (29.9)	14 (33.3)	
	≥35	93 (27.4)	4 (40.0)	78 (27.1)	11 (26.2)	
Marital status						
	Married	241 (70.9)	8 (80.0)	197 (68.4)	36 (85.7)	0.057
	Single and others	99 (29.1)	2 (20.0)	91 (31.6)	6 (14.3)	
Education level						
	None	9 (2.6)		8 (2.8)	1 (2.4)	**0.046**
	High school	117 (34.4)	8 (80.0)	96 (33.3)	13 (31.0)	
	Vocational Education and Training (VET)	14 (4.1)	1 (10.0)	10 (3.5)	3 (7.1)	
	Higher education	200 (58.8)	1 (10.0)	174 (60.4)	25 (59.5)	
Employment status						
	Employed	235 (69.1)	7 (70.0)	197 (68.4)	31 (73.8)	0.777
	Unemployed	105 (30.9)	3 (30.0)	91 (31.6)	11 (26.2)	
Ethnicity						
	Khalkh	297 (87.4)	10 (100.0)	251 (87.2)	36 (85.7)	0.458
	Others	43 (12.6)		37 (12.8)	6 (14.3)	
Parity		1.54 ± 1.25	2.00 ± 1.41	1.48 ± 1.25	1.81 ± 1.15	0.138
	Primipara	92 (27.1)	1 (10.0)	88 (30.6)	3 (7.1)	**0.003**
	Multipara	248 (72.9)	9 (90.0)	200 (69.4)	39 (92.9)	
Gravidity		3.12 ± 1.69	3.50 ± 1.26	3.03 ± 1.65	3.64 ± 1.98	0.069
	≤1	67 (19.7)	1 (10.0)	62 (21.5)	4 (9.5)	0.139
	≥2	273 (80.3)	9 (90.0)	226 (78.5)	38 (90.5)	
Smoking		11 (3.2)	2 (20.0)	7 (2.4)	2 (4.8)	**0.007**
Alcohol consumption		93 (27.4)	3 (30)	86 (29.9)	4 (9.5)	**0.022**
Pre-pregnancy						
	Weight, kg	61.10 ± 12.02	57.90 ± 12.15	59.67± 10.74	71.73 ± 14.94	**<0.001**
	Height, m	1.61 ± 0.06	1.59 ± 0.07	1.61 ± 0.06	1.63 ± 0.06	0.059
	BMI, kg/m^2^	23.48 ± 4.40	22.60 ± 4.02	23.02 ± 4.05	26.86 ± 5.29	**<0.001**
Pre-pregnancy BMI classification						
	Underweight (<18.5)	32 (9.4)	1 (10.0)	31 (10.8)		**<0.001**
	Normal (18.5–24.9)	202 (59.4)	7 (70.0)	174 (60.4)	21 (50.0)	
	Overweight (25.0–29.9)	77 (22.7)	1 (10.0)	67 (23.3)	9 (21.4)	
	Obese (≥30.0)	29 (8.5)	1 (10.0)	16 (5.5)	12 (28.6)	
Total GWG, kg		14.31 ± 6.16	8.80 ± 5.43	13.8 ± 5.60	19.10 ± 7.44	**<0.001**
GWG classification						
	Inadequate	96 (28.2)	7 (70.0)	87 (30.2)	2 (4.8)	**<0.001**
	Adequate	112 (32.9)	2 (20.0)	105 (36.5)	5 (11.9)	
	Excessive	132 (38.9)	1 (10.0)	96 (33.3)	35 (83.3)	
Maternal BMI before delivery, kg/m^2^		28.98 ± 4.97	26.12 ± 3.89	28.36 ± 4.57	33.96 ± 4.91	**<0.001**
Gestational age at delivery/days		274.21 ± 10.60	258.20 ± 20.07	274.74 ± 10.07	274.33 ± 8.15	<0.001
Gestational age at delivery classification						
	Preterm birth (<259 days/<37 weeks)	27 (7.9)	4 (40.0)	21 (7.3)	2 (4.8)	0.007
	Term birth (≥259 days/≥37 weeks)	313 (92.1)	6 (60.0)	267 (92.7)	40 (95.2)	

Notes. Data are expressed as mean ± SD and number (percentages, %). Bold values indicate statistical significance at *p* < 0.05. Abbreviations: BMI, body mass index; GWG, gestational weight gain; g, gram; kg, kilogram; SD, standard deviation.

**Table 3:** Association between pre-pregnancy BMI, GWG, and neonatal birth weight.

Model	Variable	β Coefficient (Unstandardized)	95% CI	*p*-Value
**A. Pre-pregnancy BMI Model**				
Unadjusted				
	Underweight	−125.58	−310.70 to 59.54	**0.183**
	Normal weight	0 (Reference)	-	**-**
	Overweight/Obese	166.14	48.92 to 282.84	**0.005**
Adjusted for general characteristics *				
	Underweight	−120.75	−305.57 to 64.04	0.200
	Normal weight	0 (Reference)	-	**-**
	Overweight/Obese	176.56	61.63 to 294.48	**0.003**
Adjusted for gestational age at delivery†				
	Underweight	−119	−302.1 to 63.9	0.202
	Normal weight	0 (Reference)	-	**-**
	Overweight/Obese	155.0	39.1 to 272.1	**0.009**
Adjusted for gestational characteristics ‡				
	Underweight	−73.41	−245.27 to 98.44	0.401
	Normal weight	0 (Reference)	-	**-**
	Overweight/Obese	179.39	87.07 to 277.76	**<0.001**
**B. GWG Model**				
Unadjusted				
	Inadequate	−176.86	−303.44 to −50.28	**0.006**
	Adequate	0 (Reference)	-	**-**
	Excessive	297.26	180.34 to 414.17	**<0.001**
Adjusted for general characteristics *				
	Inadequate	−184.59	−311.61 to −57.56	**0.005**
	Adequate	0 (Reference)	-	**-**
	Excessive	288.67	170.71 to 406.62	**<0.001**
Adjusted for gestational age at delivery †				
	Inadequate	−176.7	−302.3 to −51.1	**0.006**
	Adequate	0 (Reference)		
	Excessive	281.4	164.8 to 398.1	**<0.001**
Adjusted for gestational characteristics †‡				
	Inadequate	−152.75	−281.25 to −24.24	**0.020**
	Adequate	0 (Reference)	-	**-**
	Excessive	257.07	136.62 to 377.19	**<0.001**

Notes. β = unstandardized regression coefficient; CI = confidence interval. Reference categories were normal pre-pregnancy BMI and adequate GWG. Abbreviations: BMI, body mass index; GWG, gestational weight gain. * The model adjusted for general characteristics included maternal age, education level, smoking status, and alcohol use. † The model adjusted for gestational age at delivery included general characteristics (maternal age, education level, smoking status, and alcohol use) and gestational age at delivery. ‡ The model adjusted for gestational characteristics additionally included mutual adjustment between pre-pregnancy BMI and GWG (i.e., BMI was adjusted for GWG, and GWG was adjusted for BMI), as well as parity. Bold values indicate statistical significance at *p* < 0.05.

## Data Availability

The original contributions presented in this study are included in the article. Further inquiries can be directed to the corresponding authors.
